# 871. Oral Ibrexafungerp Outcomes by Fungal Disease in Patients from an Interim Analysis of a Phase 3 Open-label Study (FURI)

**DOI:** 10.1093/ofid/ofac492.064

**Published:** 2022-12-15

**Authors:** George R Thompson, Thomas King, Nkechi Azie, David A Angulo, Juergen Prattes

**Affiliations:** University of California Davis Medical Center, Sacramento, California; SCYNEXIS, Inc., Jersey City, New Jersey; SCYNEXIS, Inc., Jersey City, New Jersey; SCYNEXIS, Inc., Jersey City, New Jersey; Medical University of Graz, Graz, Steiermark, Austria

## Abstract

**Background:**

There are limited oral treatment options for patients with fungal infections who fail currently available antifungals or have an infection caused by resistant organisms. Ibrexafungerp is an investigational broad-spectrum glucan synthase inhibitor with activity against *Candida* and *Aspergillus* species, including azole- and echinocandin-resistant strains. A Phase 3 open-label, single-arm study of ibrexafungerp (FURI; NCT03059992) is ongoing for the treatment of patients intolerant of, or with fungal disease refractory to, standard antifungal therapy. We present an interim analysis of patient outcomes from the FURI study by fungal disease type.

**Methods:**

FURI patients are eligible for enrollment if they have proven or probable: severe mucocutaneous candidiasis, invasive candidiasis, chronic or invasive aspergillosis, with documented evidence of failure, intolerance, or toxicity related to a currently approved standard-of-care antifungal treatment; or patients who cannot receive approved oral antifungal options (e.g., due to susceptibility), and continued IV antifungal therapy is clinically undesirable or unfeasible.

**Results:**

An independent Data Review Committee (DRC) provided an assessment of treatment response for 113 enrolled patients in the FURI study from 27 centers in US, UK and EU treated with ibrexafungerp for mucocutaneous or invasive fungal infections from 2016–2021. Fifty-six patients (49.5%) had invasive candidiasis/candidemia, 32 (28.3%) had mucocutaneous candidiasis, 14 (12.4%) had vulvovaginal candidiasis (VVC), and 11 (9.7%) patients had aspergillosis.

Upon DRC review, the percent of patients with complete or partial response, or for VVC, clinical improvement (defined as vulvovaginal signs and symptoms score ≤ 1) was 58.4%; stable disease was 23.9%; and 11.5% had disease progression (including 2 VVC patients not meeting the criteria for clinical improvement). There was 1 death due to underlying causes, and 6 outcomes were indeterminate. The table presents outcomes by disease type.

**Conclusion:**

Analysis of 113 patients from the FURI study indicates that oral ibrexafungerp provides a favorable therapeutic response in patients with challenging fungal disease and limited treatment options.

**Disclosures:**

**George R. Thompson, III, MD**, Amplyx: Advisor/Consultant|Amplyx: Grant/Research Support|Astellas: Advisor/Consultant|Astellas: Grant/Research Support|Cidara: Advisor/Consultant|Cidara: Grant/Research Support|F2G: Advisor/Consultant|F2G: Grant/Research Support|Merck: Grant/Research Support|Pfizer: DSMB|Scynexis: Advisor/Consultant|Scynexis: Grant/Research Support **Thomas King, MS MPH**, SCYNEXIS, Inc.: employee|SCYNEXIS, Inc.: Stocks/Bonds **Nkechi Azie, MD MBA FIDSA**, SCYNEXIS, Inc.: employee|SCYNEXIS, Inc.: Stocks/Bonds **David A. Angulo, MD**, SCYNEXIS, Inc.: employee|SCYNEXIS, Inc.: Stocks/Bonds.

